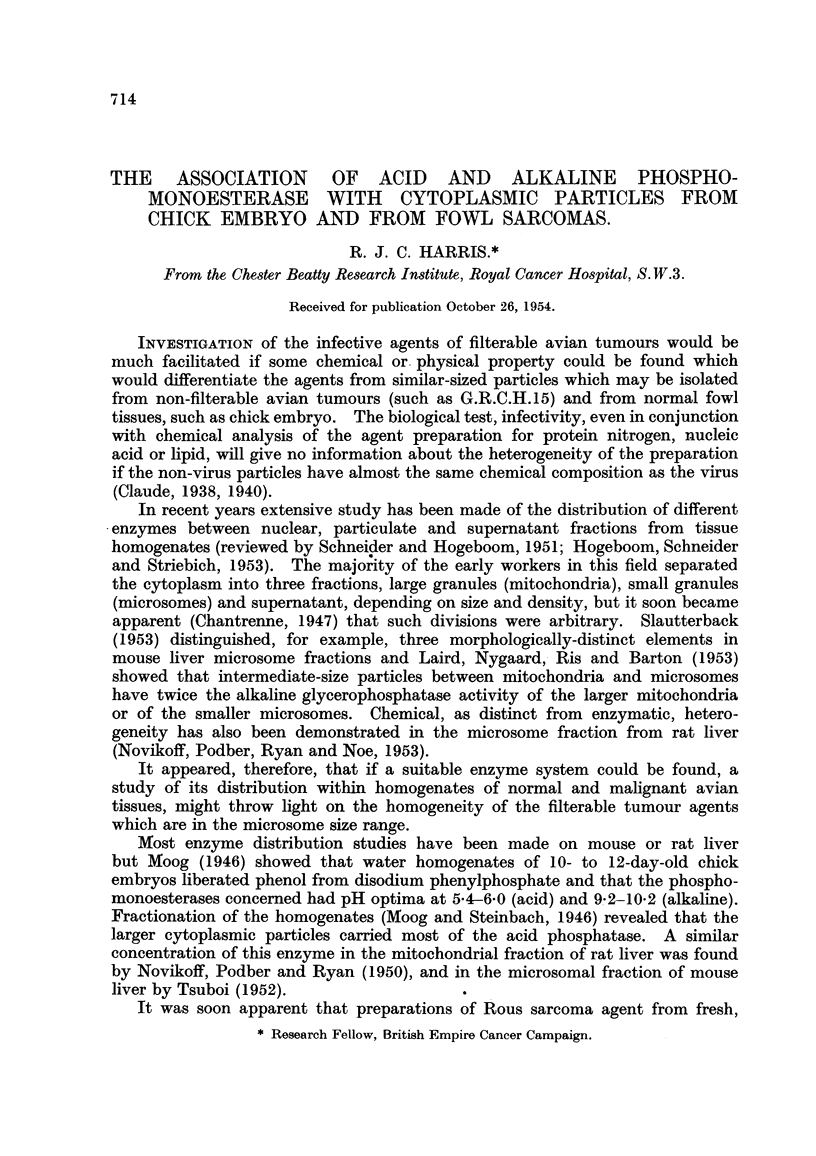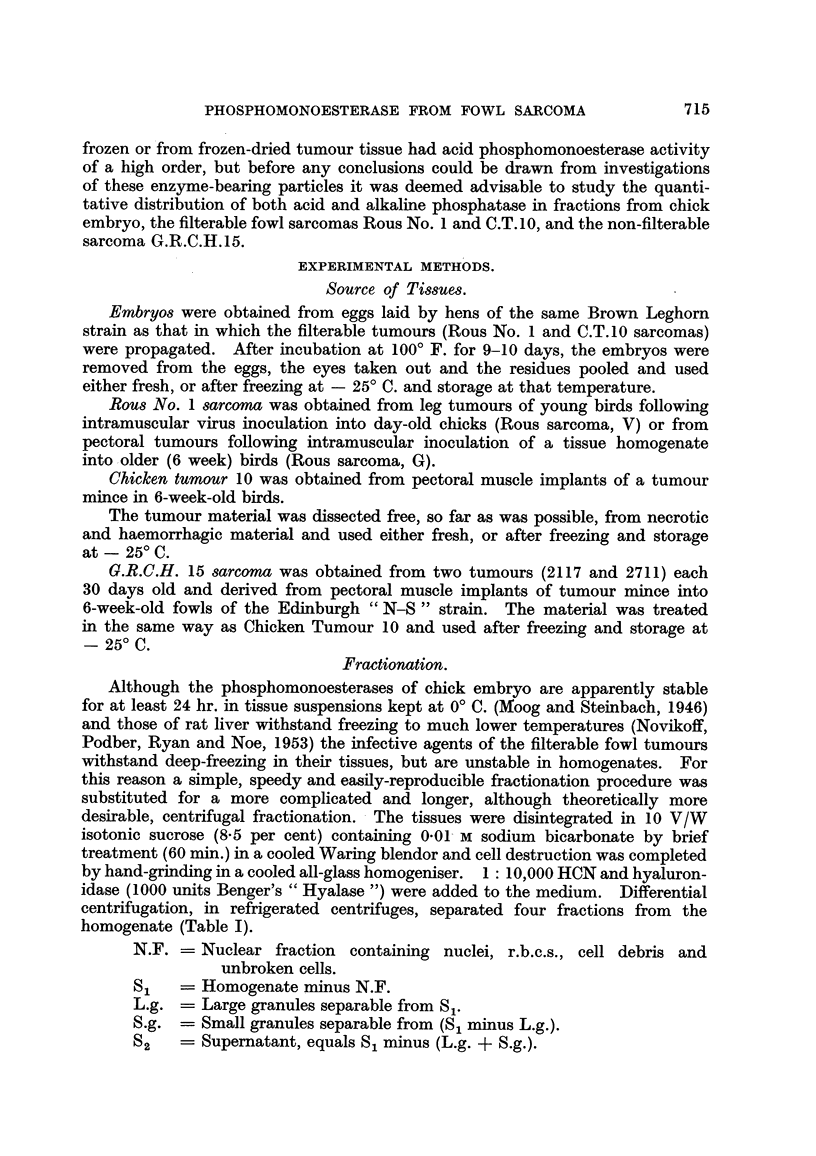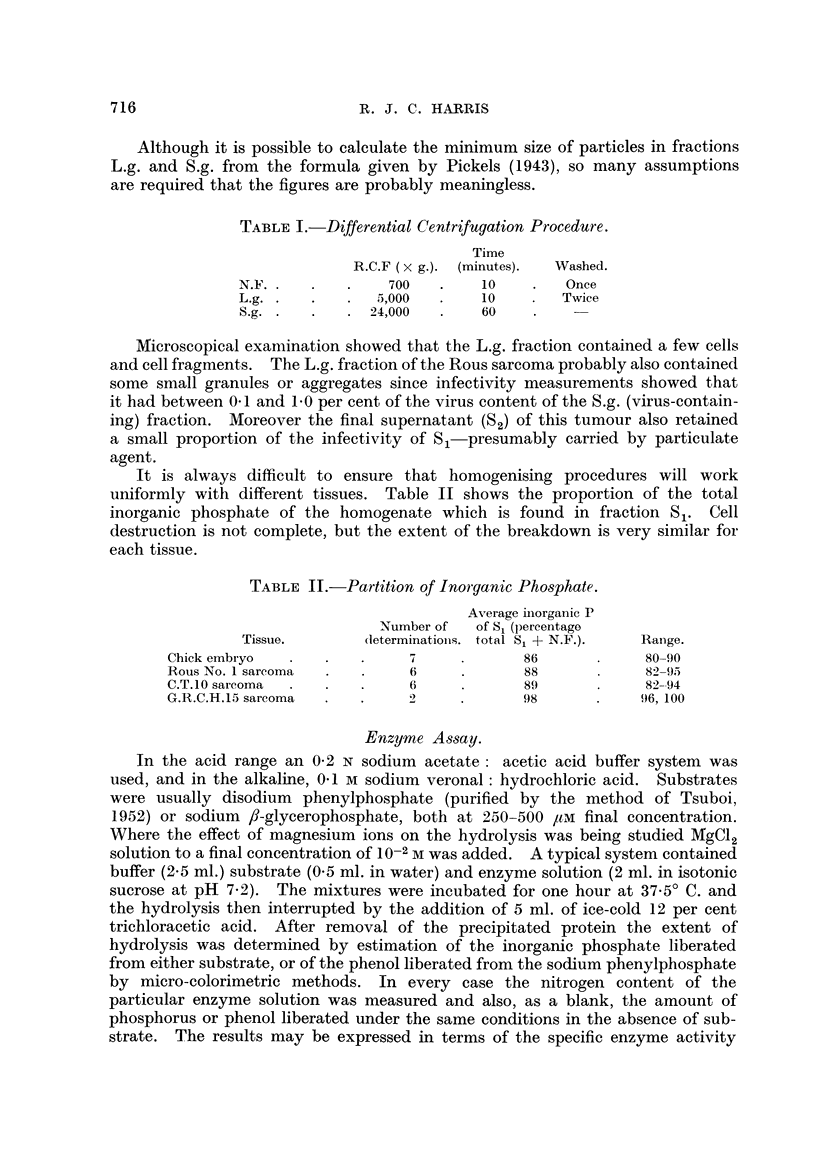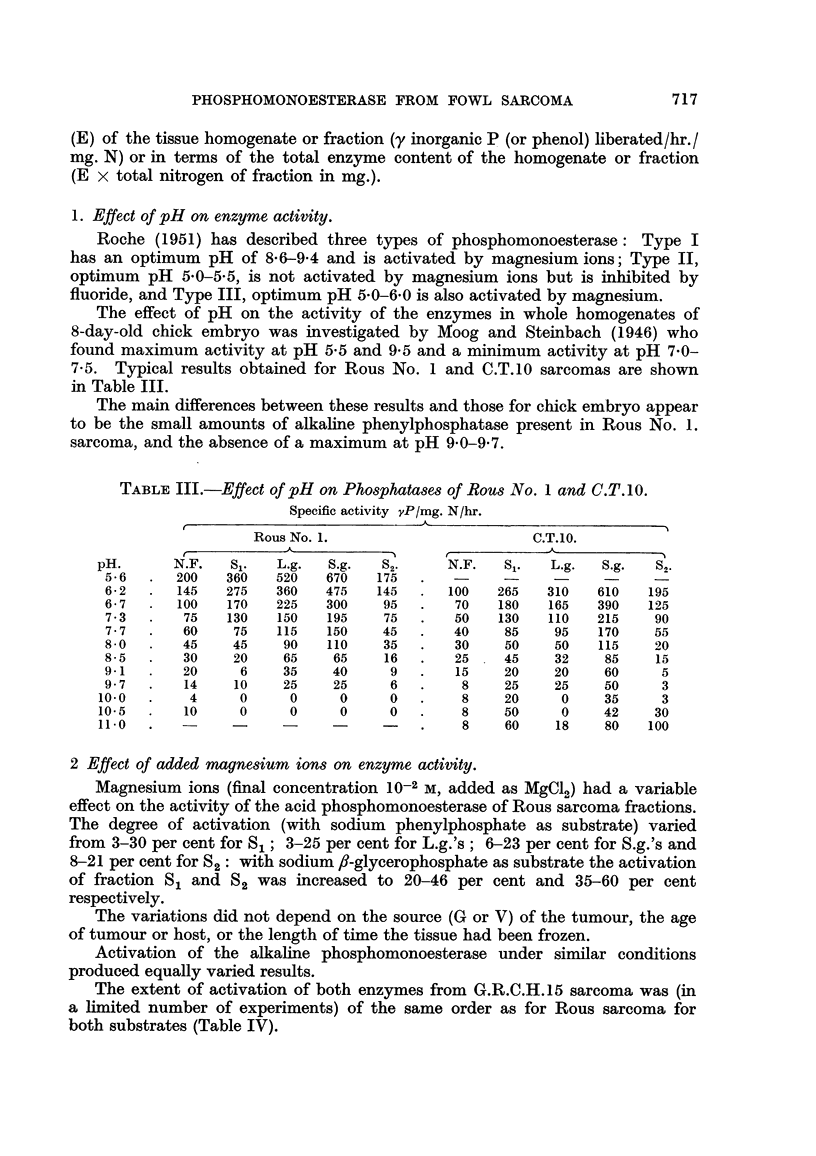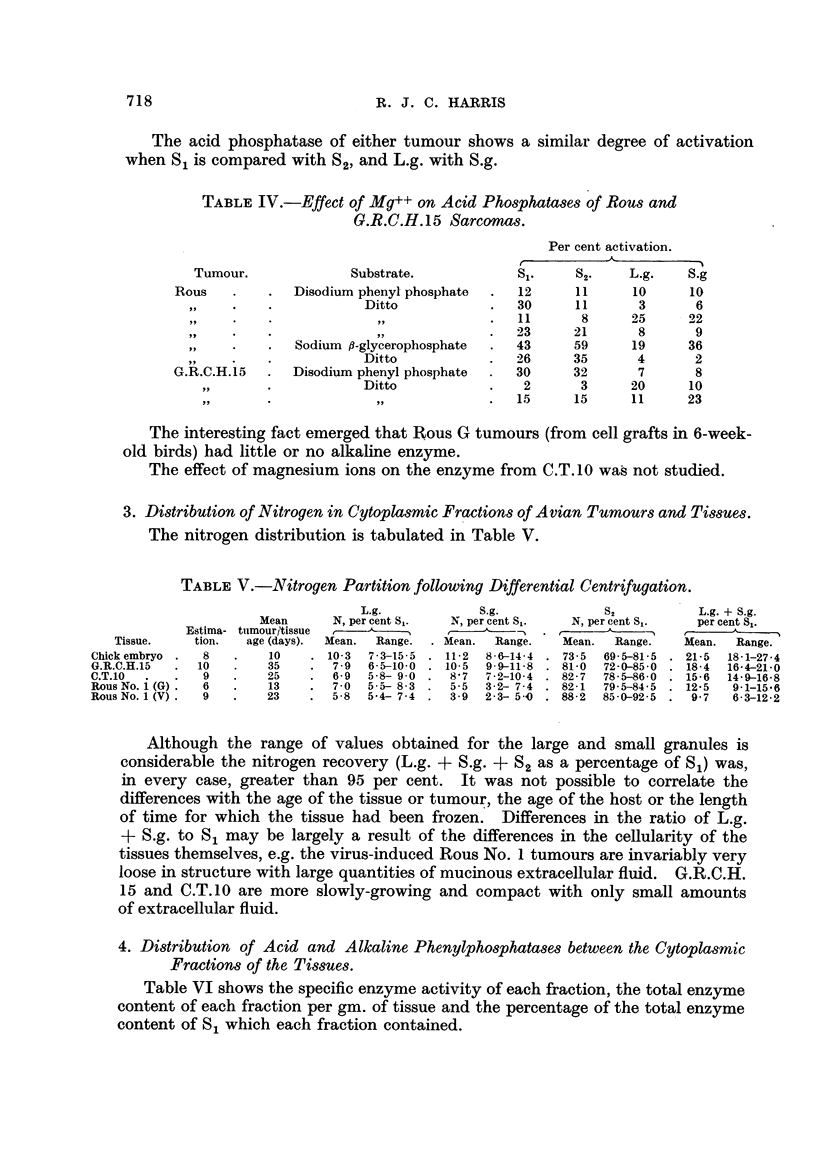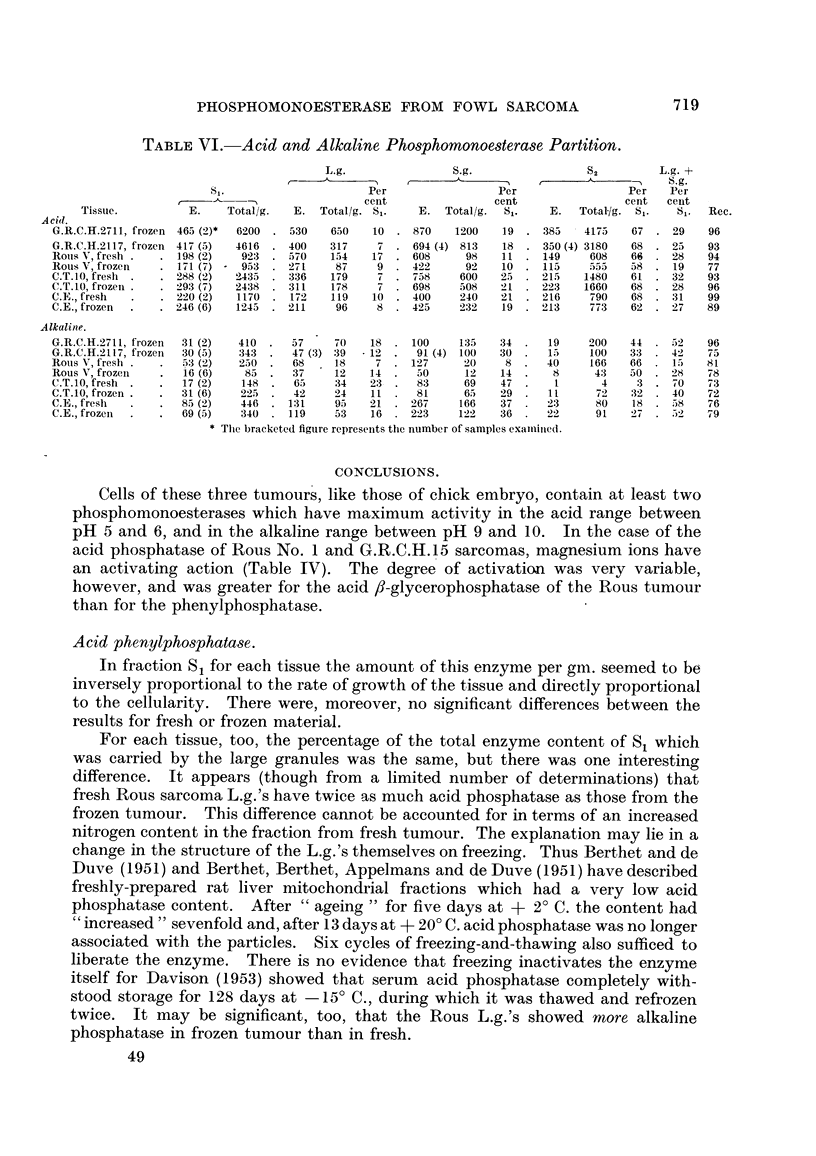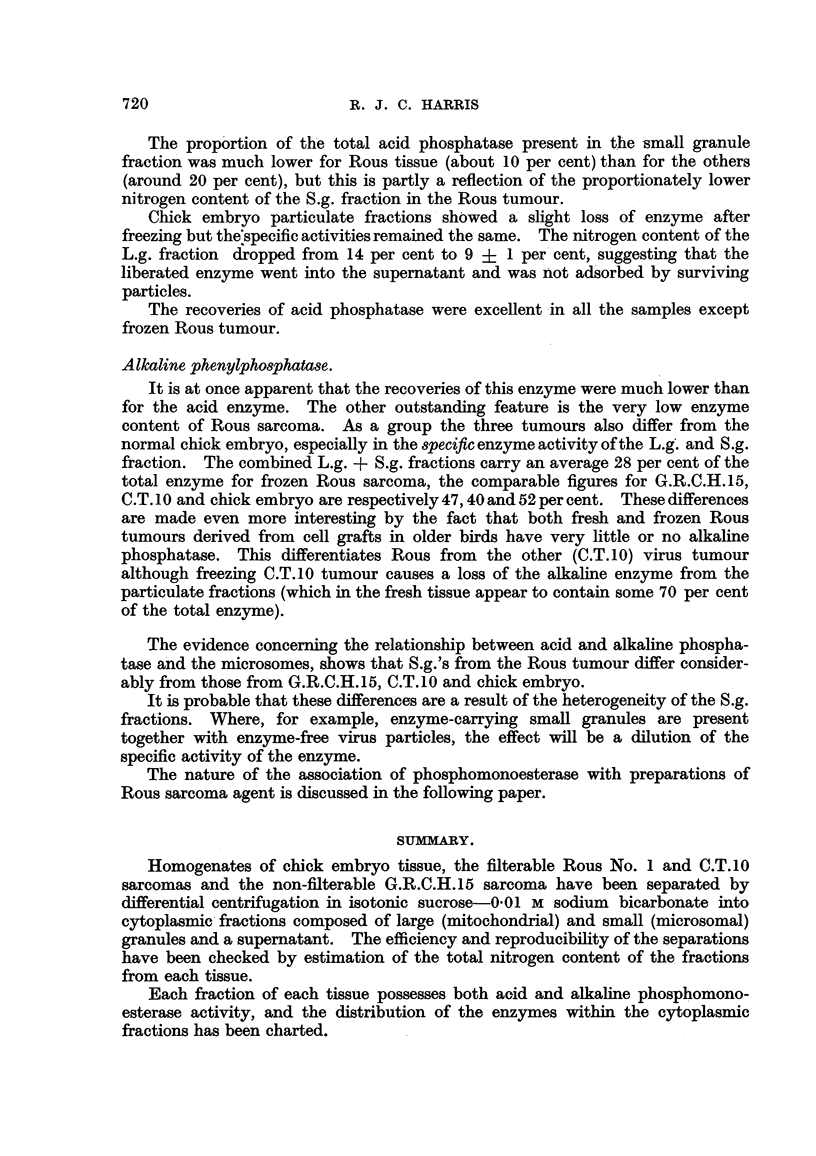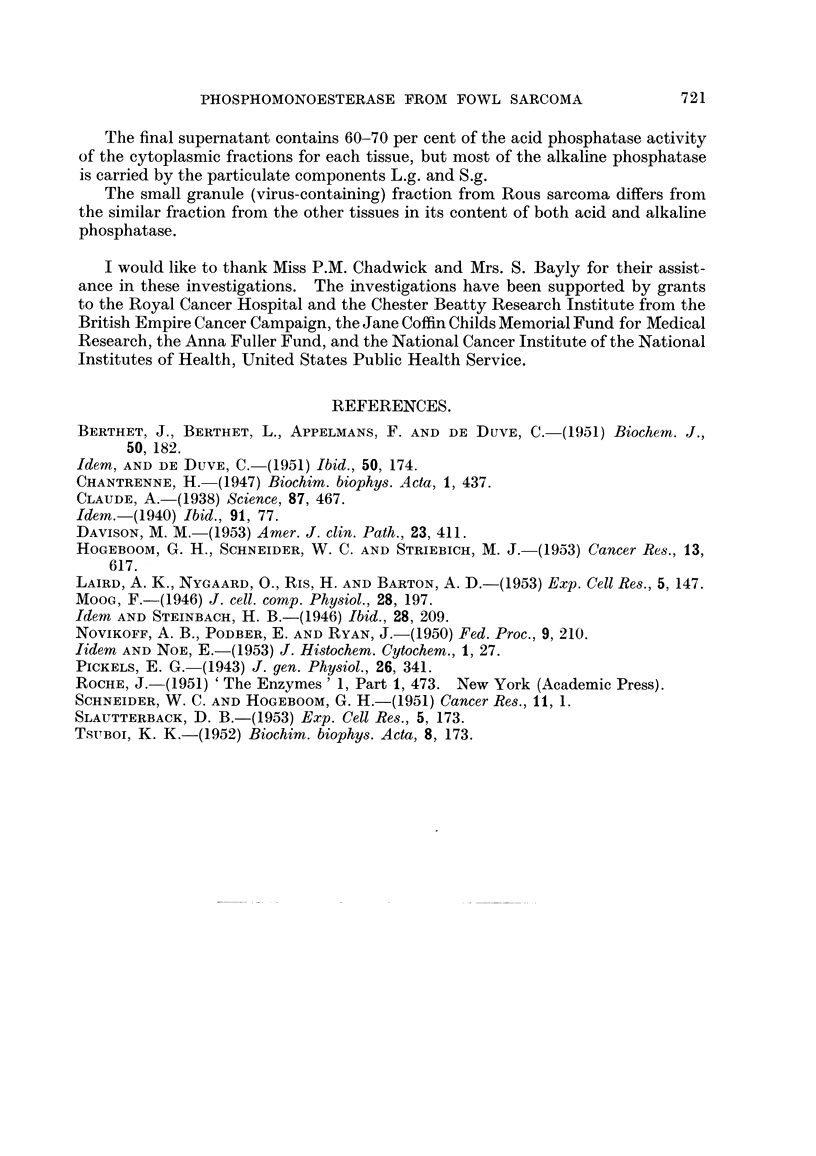# The Association of Acid and Alkaline Phosphomonoesterase with Cytoplasmic Particles from Chick Embryo and from Fowl Sarcomas

**DOI:** 10.1038/bjc.1954.79

**Published:** 1954-12

**Authors:** R. J. C. Harris


### THE ASSOCIATION OF ACID AND ALKALINE PHOSPHO-MONOESTERASE WITH CYTOPLASMIC PARTICLES FROM CHICK EMBRYO AND FROM FOWL SARCOMAS.

R. J. C. HARRIS.*

*From the Chester Beatty Research Institute, Royal Cancer Hospital, S.W.3.*

Received for publication October 26, 1954.

INVESTIGATION of the infective agents of filterable avian tumours would be much facilitated if some chemical or physical property could be found which would differentiate the agents from similar-sized particles which may be isolated from non-filterable avian tumours (such as G.R.C.H.15) and from normal fowl tissues, such as chick embryo. The biological test, infectivity, even in conjunction with chemical analysis of the agent preparation for protein nitrogen, nucleic acid or lipid, will give no information about the heterogeneity of the preparation if the non-virus particles have almost the same chemical composition as the virus (Claude, 1938, 1940).

In recent years extensive study has been made of the distribution of different enzymes between nuclear, particulate and supernatant fractions from tissue homogenates (reviewed by Schneider and Hogeboom, 1951; Hogeboom, Schneider and Striebich, 1953). The majority of the early workers in this field separated the cytoplasm into three fractions, large granules (mitochondria), small granules (microsomes) and supernatant, depending on size and density, but it soon became apparent (Chantrenne, 1947) that such divisions were arbitrary. Slautterback (1953) distinguished, for example, three morphologically-distinct elements in mouse liver microsome fractions and Laird, Nygaard, Ris and Barton (1953) showed that intermediate-size particles between mitochondria and microsomes have twice the alkaline glycerophosphatase activity of the larger mitochondria or of the smaller microsomes. Chemical, as distinct from enzymatic, heterogeneity has also been demonstrated in the microsome fraction from rat liver (Novikoff, Podber, Ryan and Noe, 1953).

It appeared, therefore, that if a suitable enzyme system could be found, a study of its distribution within homogenates of normal and malignant avian tissues, might throw light on the homogeneity of the filterable tumour agents which are in the microsome size range.

Most enzyme distribution studies have been made on mouse or rat liver but Moog (1946) showed that water homogenates of 10- to 12-day-old chick embryos liberated phenol from disodium phenylphosphate and that the phosphomonoesterases concerned had pH optima at 5·4–6·0 (acid) and 9·2–10·2 (alkaline). Fractionation of the homogenates (Moog and Steinbach, 1946) revealed that the larger cytoplasmic particles carried most of the acid phosphatase. A similar concentration of this enzyme in the mitochondrial fraction of rat liver was found by Novikoff, Podber and Ryan (1950), and in the microsomal fraction of mouse liver by Tsuboi (1952).

It was soon apparent that preparations of Rous sarcoma agent from fresh,

* Research Fellow, British Empire Cancer Campaign.

frozen or from frozen-dried tumour tissue had acid phosphomonoesterase activity of a high order, but before any conclusions could be drawn from investigations of these enzyme-bearing particles it was deemed advisable to study the quantitative distribution of both acid and alkaline phosphatase in fractions from chick embryo, the filterable fowl sarcomas Rous No. 1 and C.T.10, and the non-filterable sarcoma G.R.C.H.15.

### EXPERIMENTAL METHODS.

### *Source of Tissues.*

*Embryos* were obtained from eggs laid by hens of the same Brown Leghorn strain as that in which the filterable tumours (Rous No. 1 and C.T.10 sarcomas) were propagated. After incubation at 100° F. for 9–10 days, the embryos were removed from the eggs, the eyes taken out and the residues pooled and used either fresh, or after freezing at — 25° C. and storage at that temperature.

*Rous No. 1 sarcoma* was obtained from leg tumours of young birds following intramuscular virus inoculation into day-old chicks (Rous sarcoma, V) or from pectoral tumours following intramuscular inoculation of a tissue homogenate into older (6 week) birds (Rous sarcoma, G).

*Chicken tumour* 10 was obtained from pectoral muscle implants of a tumour mince in 6-week-old birds.

The tumour material was dissected free, so far as was possible, from necrotic and haemorrhagic material and used either fresh, or after freezing and storage at — 25° C.

*G.R.C.H.* 15 *sarcoma* was obtained from two tumours (2117 and 2711) each 30 days old and derived from pectoral muscle implants of tumour mince into 6-week-old fowls of the Edinburgh " N–S " strain. The material was treated in the same way as Chicken Tumour 10 and used after freezing and storage at — 25° C.

### *Fractionation.*

Although the phosphomonoesterases of chick embryo are apparently stable for at least 24 hr. in tissue suspensions kept at 0° C. (Moog and Steinbach, 1946) and those of rat liver withstand freezing to much lower temperatures (Novikoff, Podber, Ryan and Noe, 1953) the infective agents of the filterable fowl tumours withstand deep-freezing in their tissues, but are unstable in homogenates. For this reason a simple, speedy and easily-reproducible fractionation procedure was substituted for a more complicated and longer, although theoretically more desirable, centrifugal fractionation. The tissues were disintegrated in 10 V/W isotonic sucrose (8·5 per cent) containing 0·01 M sodium bicarbonate by brief treatment (60 min.) in a cooled Waring blendor and cell destruction was completed by hand-grinding in a cooled all-glass homogeniser. 1 : 10,000 HCN and hyaluronidase (1000 units Benger's " Hyalase ") were added to the medium. Differential centrifugation, in refrigerated centrifuges, separated four fractions from the homogenate (Table I).

N.F. = Nuclear fraction containing nuclei, r.b.c.s., cell debris and unbroken cells.
$S_1$ = Homogenate minus N.F.
L.g. = Large granules separable from $S_1$.
S.g. = Small granules separable from ($S_1$ minus L.g.).
$S_2$ = Supernatant, equals $S_1$ minus (L.g. + S.g.).

Although it is possible to calculate the minimum size of particles in fractions L.g. and S.g. from the formula given by Pickels (1943), so many assumptions are required that the figures are probably meaningless.

TABLE I.—*Differential Centrifugation Procedure.*

| | R.C.F ($\times$ g.). | Time (minutes). | Washed. |
|---|---|---|---|
| N.F. | 700 | 10 | Once |
| L.g. | 5,000 | 10 | Twice |
| S.g. | 24,000 | 60 | — |

Microscopical examination showed that the L.g. fraction contained a few cells and cell fragments. The L.g. fraction of the Rous sarcoma probably also contained some small granules or aggregates since infectivity measurements showed that it had between 0·1 and 1·0 per cent of the virus content of the S.g. (virus-containing) fraction. Moreover the final supernatant ($S_2$) of this tumour also retained a small proportion of the infectivity of $S_1$—presumably carried by particulate agent.

It is always difficult to ensure that homogenising procedures will work uniformly with different tissues. Table II shows the proportion of the total inorganic phosphate of the homogenate which is found in fraction $S_1$. Cell destruction is not complete, but the extent of the breakdown is very similar for each tissue.

TABLE II.—*Partition of Inorganic Phosphate.*

| Tissue. | Number of determinations. | Average inorganic P of $S_1$ (percentage total $S_1$ + N.F.). | Range. |
|---|---|---|---|
| Chick embryo | 7 | 86 | 80–90 |
| Rous No. 1 sarcoma | 6 | 88 | 82–95 |
| C.T.10 sarcoma | 6 | 89 | 82–94 |
| G.R.C.H.15 sarcoma | 2 | 98 | 96, 100 |

*Enzyme Assay.*

In the acid range an 0·2 N sodium acetate : acetic acid buffer system was used, and in the alkaline, 0·1 M sodium veronal : hydrochloric acid. Substrates were usually disodium phenylphosphate (purified by the method of Tsuboi, 1952) or sodium $\beta$-glycerophosphate, both at 250–500 $\mu$M final concentration. Where the effect of magnesium ions on the hydrolysis was being studied $MgCl_2$ solution to a final concentration of $10^{-2}$ M was added. A typical system contained buffer (2·5 ml.) substrate (0·5 ml. in water) and enzyme solution (2 ml. in isotonic sucrose at pH 7·2). The mixtures were incubated for one hour at 37·5° C. and the hydrolysis then interrupted by the addition of 5 ml. of ice-cold 12 per cent trichloracetic acid. After removal of the precipitated protein the extent of hydrolysis was determined by estimation of the inorganic phosphate liberated from either substrate, or of the phenol liberated from the sodium phenylphosphate by micro-colorimetric methods. In every case the nitrogen content of the particular enzyme solution was measured and also, as a blank, the amount of phosphorus or phenol liberated under the same conditions in the absence of substrate. The results may be expressed in terms of the specific enzyme activity

(E) of the tissue homogenate or fraction ($\gamma$ inorganic P (or phenol) liberated/hr./mg. N) or in terms of the total enzyme content of the homogenate or fraction (E $\times$ total nitrogen of fraction in mg.).

1. *Effect of pH on enzyme activity.*

Roche (1951) has described three types of phosphomonoesterase: Type I has an optimum pH of 8·6–9·4 and is activated by magnesium ions; Type II, optimum pH 5·0–5·5, is not activated by magnesium ions but is inhibited by fluoride, and Type III, optimum pH 5·0–6·0 is also activated by magnesium.

The effect of pH on the activity of the enzymes in whole homogenates of 8-day-old chick embryo was investigated by Moog and Steinbach (1946) who found maximum activity at pH 5·5 and 9·5 and a minimum activity at pH 7·0–7·5. Typical results obtained for Rous No. 1 and C.T.10 sarcomas are shown in Table III.

The main differences between these results and those for chick embryo appear to be the small amounts of alkaline phenylphosphatase present in Rous No. 1. sarcoma, and the absence of a maximum at pH 9·0–9·7.

TABLE III.—*Effect of pH on Phosphatases of Rous No.* 1 *and C.T.*10.

Specific activity $\gamma P$/mg. N/hr.

| | Rous No. 1. | | | | | C.T.10. | | | | |
|---|---|---|---|---|---|---|---|---|---|---|
| pH. | N.F. | $S_1$. | L.g. | S.g. | $S_2$. | N.F. | $S_1$. | L.g. | S.g. | $S_2$. |
| 5·6 | 200 | 360 | 520 | 670 | 175 | — | — | — | — | — |
| 6·2 | 145 | 275 | 360 | 475 | 145 | 100 | 265 | 310 | 610 | 195 |
| 6·7 | 100 | 170 | 225 | 300 | 95 | 70 | 180 | 165 | 390 | 125 |
| 7·3 | 75 | 130 | 150 | 195 | 75 | 50 | 130 | 110 | 215 | 90 |
| 7·7 | 60 | 75 | 115 | 150 | 45 | 40 | 85 | 95 | 170 | 55 |
| 8·0 | 45 | 45 | 90 | 110 | 35 | 30 | 50 | 50 | 115 | 20 |
| 8·5 | 30 | 20 | 65 | 65 | 16 | 25 | 45 | 32 | 85 | 15 |
| 9·1 | 20 | 6 | 35 | 40 | 9 | 15 | 20 | 20 | 60 | 5 |
| 9·7 | 14 | 10 | 25 | 25 | 6 | 8 | 25 | 25 | 50 | 3 |
| 10·0 | 4 | 0 | 0 | 0 | 0 | 8 | 20 | 0 | 35 | 3 |
| 10·5 | 10 | 0 | 0 | 0 | 0 | 8 | 50 | 0 | 42 | 30 |
| 11·0 | — | — | — | — | — | 8 | 60 | 18 | 80 | 100 |

2 *Effect of added magnesium ions on enzyme activity.*

Magnesium ions (final concentration $10^{-2}$ M, added as $MgCl_2$) had a variable effect on the activity of the acid phosphomonoesterase of Rous sarcoma fractions. The degree of activation (with sodium phenylphosphate as substrate) varied from 3–30 per cent for $S_1$; 3–25 per cent for L.g.'s; 6–23 per cent for S.g.'s and 8–21 per cent for $S_2$: with sodium $\beta$-glycerophosphate as substrate the activation of fraction $S_1$ and $S_2$ was increased to 20–46 per cent and 35–60 per cent respectively.

The variations did not depend on the source (G or V) of the tumour, the age of tumour or host, or the length of time the tissue had been frozen.

Activation of the alkaline phosphomonoesterase under similar conditions produced equally varied results.

The extent of activation of both enzymes from G.R.C.H.15 sarcoma was (in a limited number of experiments) of the same order as for Rous sarcoma for both substrates (Table IV).

The acid phosphatase of either tumour shows a similar degree of activation when $S_1$ is compared with $S_2$, and L.g. with S.g.

TABLE IV.—*Effect of $Mg^{++}$ on Acid Phosphatases of Rous and G.R.C.H.*15 *Sarcomas.*

| Tumour. | Substrate. | Per cent activation. $S_1$. | $S_2$. | L.g. | S.g |
|---|---|---|---|---|---|
| Rous | Disodium phenyl phosphate | 12 | 11 | 10 | 10 |
| ,, | Ditto | 30 | 11 | 3 | 6 |
| ,, | ,, | 11 | 8 | 25 | 22 |
| ,, | ,, | 23 | 21 | 8 | 9 |
| ,, | Sodium β-glycerophosphate | 43 | 59 | 19 | 36 |
| ,, | Ditto | 26 | 35 | 4 | 2 |
| G.R.C.H.15 | Disodium phenyl phosphate | 30 | 32 | 7 | 8 |
| ,, | Ditto | 2 | 3 | 20 | 10 |
| ,, | ,, | 15 | 15 | 11 | 23 |

The interesting fact emerged that Rous G tumours (from cell grafts in 6-week-old birds) had little or no alkaline enzyme.

The effect of magnesium ions on the enzyme from C.T.10 was not studied.

3. *Distribution of Nitrogen in Cytoplasmic Fractions of Avian Tumours and Tissues.*

The nitrogen distribution is tabulated in Table V.

TABLE V.—*Nitrogen Partition following Differential Centrifugation.*

| Tissue. | Estimation. | Mean tumour/tissue age (days). | L.g. N, per cent $S_1$. Mean. | Range. | S.g. N, per cent $S_1$. Mean. | Range. | $S_2$ N, per cent $S_1$. Mean. | Range. | L.g. + S.g. per cent $S_1$. Mean. | Range. |
|---|---|---|---|---|---|---|---|---|---|---|
| Chick embryo | 8 | 10 | 10·3 | 7·3–15·5 | 11·2 | 8·6–14·4 | 73·5 | 69·5–81·5 | 21·5 | 18·1–27·4 |
| G.R.C.H.15 | 10 | 35 | 7·9 | 6·5–10·0 | 10·5 | 9·9–11·8 | 81·0 | 72·0–85·0 | 18·4 | 16·4–21·0 |
| C.T.10 | 9 | 25 | 6·9 | 5·8– 9·0 | 8·7 | 7·2–10·4 | 82·7 | 78·5–86·0 | 15·6 | 14·9–16·8 |
| Rous No. 1 (G) | 6 | 13 | 7·0 | 5·5– 8·3 | 5·5 | 3·2– 7·4 | 82·1 | 79·5–84·5 | 12·5 | 9·1–15·6 |
| Rous No. 1 (V) | 9 | 23 | 5·8 | 5·4– 7·4 | 3·9 | 2·3– 5·0 | 88·2 | 85·0–92·5 | 9·7 | 6·3–12·2 |

Although the range of values obtained for the large and small granules is considerable the nitrogen recovery (L.g. + S.g. + $S_2$ as a percentage of $S_1$) was, in every case, greater than 95 per cent. It was not possible to correlate the differences with the age of the tissue or tumour, the age of the host or the length of time for which the tissue had been frozen. Differences in the ratio of L.g. + S.g. to $S_1$ may be largely a result of the differences in the cellularity of the tissues themselves, e.g. the virus-induced Rous No. 1 tumours are invariably very loose in structure with large quantities of mucinous extracellular fluid. G.R.C.H. 15 and C.T.10 are more slowly-growing and compact with only small amounts of extracellular fluid.

4. *Distribution of Acid and Alkaline Phenylphosphatases between the Cytoplasmic Fractions of the Tissues.*

Table VI shows the specific enzyme activity of each fraction, the total enzyme content of each fraction per gm. of tissue and the percentage of the total enzyme content of $S_1$ which each fraction contained.

TABLE VI.—*Acid and Alkaline Phosphomonoesterase Partition.*

| Tissue. | $S_1$. E. | $S_1$. Total/g. | L.g. E. | L.g. Total/g. | L.g. Per cent $S_1$. | S.g. E. | S.g. Total/g. | S.g. Per cent $S_1$. | $S_2$ E. | $S_2$ Total/g. | $S_2$ Per cent $S_1$. | L.g. + S.g. Per cent $S_1$. | Rec. |
|---|---|---|---|---|---|---|---|---|---|---|---|---|---|
| *Acid.* | | | | | | | | | | | | | |
| G.R.C.H.2711, frozen | 465 (2)* | 6200 | 530 | 650 | 10 | 870 | 1200 | 19 | 385 | 4175 | 67 | 29 | 96 |
| G.R.C.H.2117, frozen | 417 (5) | 4616 | 400 | 317 | 7 | 694 (4) | 813 | 18 | 350 (4) | 3180 | 68 | 25 | 93 |
| Rous V, fresh | 198 (2) | 923 | 570 | 154 | 17 | 608 | 98 | 11 | 149 | 608 | 66 | 28 | 94 |
| Rous V, frozen | 171 (7) | 953 | 271 | 87 | 9 | 422 | 92 | 10 | 115 | 555 | 58 | 19 | 77 |
| C.T.10, fresh | 288 (2) | 2435 | 336 | 179 | 7 | 758 | 600 | 25 | 215 | 1480 | 61 | 32 | 93 |
| C.T.10, frozen | 293 (7) | 2438 | 311 | 178 | 7 | 698 | 508 | 21 | 223 | 1660 | 68 | 28 | 96 |
| C.E., fresh | 220 (2) | 1170 | 172 | 119 | 10 | 400 | 240 | 21 | 216 | 790 | 68 | 31 | 99 |
| C.E., frozen | 246 (6) | 1245 | 211 | 96 | 8 | 425 | 232 | 19 | 213 | 773 | 62 | 27 | 89 |
| *Alkaline.* | | | | | | | | | | | | | |
| G.R.C.H.2711, frozen | 31 (2) | 410 | 57 | 70 | 18 | 100 | 135 | 34 | 19 | 200 | 44 | 52 | 96 |
| G.R.C.H.2117, frozen | 30 (5) | 343 | 47 (3) | 39 | 12 | 91 (4) | 100 | 30 | 15 | 100 | 33 | 42 | 75 |
| Rous V, fresh | 53 (2) | 250 | 68 | 18 | 7 | 127 | 20 | 8 | 40 | 166 | 66 | 15 | 81 |
| Rous V, frozen | 16 (6) | 85 | 37 | 12 | 14 | 50 | 12 | 14 | 8 | 43 | 50 | 28 | 78 |
| C.T.10, fresh | 17 (2) | 148 | 65 | 34 | 23 | 83 | 69 | 47 | 1 | 4 | 3 | 70 | 73 |
| C.T.10, frozen | 31 (6) | 225 | 42 | 24 | 11 | 81 | 65 | 29 | 11 | 72 | 32 | 40 | 72 |
| C.E., fresh | 85 (2) | 446 | 131 | 95 | 21 | 267 | 166 | 37 | 23 | 80 | 18 | 58 | 76 |
| C.E., frozen | 69 (5) | 340 | 119 | 53 | 16 | 223 | 122 | 36 | 22 | 91 | 27 | 52 | 79 |

* The bracketed figure represents the number of samples examined.

### CONCLUSIONS.

Cells of these three tumours, like those of chick embryo, contain at least two phosphomonoesterases which have maximum activity in the acid range between pH 5 and 6, and in the alkaline range between pH 9 and 10. In the case of the acid phosphatase of Rous No. 1 and G.R.C.H.15 sarcomas, magnesium ions have an activating action (Table IV). The degree of activation was very variable, however, and was greater for the acid $\beta$-glycerophosphatase of the Rous tumour than for the phenylphosphatase.

*Acid phenylphosphatase.*

In fraction $S_1$ for each tissue the amount of this enzyme per gm. seemed to be inversely proportional to the rate of growth of the tissue and directly proportional to the cellularity. There were, moreover, no significant differences between the results for fresh or frozen material.

For each tissue, too, the percentage of the total enzyme content of $S_1$ which was carried by the large granules was the same, but there was one interesting difference. It appears (though from a limited number of determinations) that fresh Rous sarcoma L.g.'s have twice as much acid phosphatase as those from the frozen tumour. This difference cannot be accounted for in terms of an increased nitrogen content in the fraction from fresh tumour. The explanation may lie in a change in the structure of the L.g.'s themselves on freezing. Thus Berthet and de Duve (1951) and Berthet, Berthet, Appelmans and de Duve (1951) have described freshly-prepared rat liver mitochondrial fractions which had a very low acid phosphatase content. After "ageing" for five days at + 2° C. the content had "increased" sevenfold and, after 13 days at + 20° C. acid phosphatase was no longer associated with the particles. Six cycles of freezing-and-thawing also sufficed to liberate the enzyme. There is no evidence that freezing inactivates the enzyme itself for Davison (1953) showed that serum acid phosphatase completely withstood storage for 128 days at —15° C., during which it was thawed and refrozen twice. It may be significant, too, that the Rous L.g.'s showed *more* alkaline phosphatase in frozen tumour than in fresh.

The proportion of the total acid phosphatase present in the small granule fraction was much lower for Rous tissue (about 10 per cent) than for the others (around 20 per cent), but this is partly a reflection of the proportionately lower nitrogen content of the S.g. fraction in the Rous tumour.

Chick embryo particulate fractions showed a slight loss of enzyme after freezing but the specific activities remained the same. The nitrogen content of the L.g. fraction dropped from 14 per cent to 9 $\pm$ 1 per cent, suggesting that the liberated enzyme went into the supernatant and was not adsorbed by surviving particles.

The recoveries of acid phosphatase were excellent in all the samples except frozen Rous tumour.

*Alkaline phenylphosphatase.*

It is at once apparent that the recoveries of this enzyme were much lower than for the acid enzyme. The other outstanding feature is the very low enzyme content of Rous sarcoma. As a group the three tumours also differ from the normal chick embryo, especially in the *specific* enzyme activity of the L.g. and S.g. fraction. The combined L.g. + S.g. fractions carry an average 28 per cent of the total enzyme for frozen Rous sarcoma, the comparable figures for G.R.C.H.15, C.T.10 and chick embryo are respectively 47, 40 and 52 per cent. These differences are made even more interesting by the fact that both fresh and frozen Rous tumours derived from cell grafts in older birds have very little or no alkaline phosphatase. This differentiates Rous from the other (C.T.10) virus tumour although freezing C.T.10 tumour causes a loss of the alkaline enzyme from the particulate fractions (which in the fresh tissue appear to contain some 70 per cent of the total enzyme).

The evidence concerning the relationship between acid and alkaline phosphatase and the microsomes, shows that S.g.'s from the Rous tumour differ considerably from those from G.R.C.H.15, C.T.10 and chick embryo.

It is probable that these differences are a result of the heterogeneity of the S.g. fractions. Where, for example, enzyme-carrying small granules are present together with enzyme-free virus particles, the effect will be a dilution of the specific activity of the enzyme.

The nature of the association of phosphomonoesterase with preparations of Rous sarcoma agent is discussed in the following paper.

### SUMMARY.

Homogenates of chick embryo tissue, the filterable Rous No. 1 and C.T.10 sarcomas and the non-filterable G.R.C.H.15 sarcoma have been separated by differential centrifugation in isotonic sucrose—0·01 M sodium bicarbonate into cytoplasmic fractions composed of large (mitochondrial) and small (microsomal) granules and a supernatant. The efficiency and reproducibility of the separations have been checked by estimation of the total nitrogen content of the fractions from each tissue.

Each fraction of each tissue possesses both acid and alkaline phosphomonoesterase activity, and the distribution of the enzymes within the cytoplasmic fractions has been charted.

The final supernatant contains 60–70 per cent of the acid phosphatase activity of the cytoplasmic fractions for each tissue, but most of the alkaline phosphatase is carried by the particulate components L.g. and S.g.

The small granule (virus-containing) fraction from Rous sarcoma differs from the similar fraction from the other tissues in its content of both acid and alkaline phosphatase.

I would like to thank Miss P.M. Chadwick and Mrs. S. Bayly for their assistance in these investigations. The investigations have been supported by grants to the Royal Cancer Hospital and the Chester Beatty Research Institute from the British Empire Cancer Campaign, the Jane Coffin Childs Memorial Fund for Medical Research, the Anna Fuller Fund, and the National Cancer Institute of the National Institutes of Health, United States Public Health Service.

### REFERENCES.

Berthet, J., Berthet, L., Appelmans, F. and de Duve, C.—(1951) *Biochem. J.*, **50**, 182.
*Idem*, and de Duve, C.—(1951) *Ibid.*, **50**, 174.
Chantrenne, H.—(1947) *Biochim. biophys. Acta*, **1**, 437.
Claude, A.—(1938) *Science*, **87**, 467.
*Idem.*—(1940) *Ibid.*, **91**, 77.
Davison, M. M.—(1953) *Amer. J. clin. Path.*, **23**, 411.
Hogeboom, G. H., Schneider, W. C. and Striebich, M. J.—(1953) *Cancer Res.*, **13**, 617.
Laird, A. K., Nygaard, O., Ris, H. and Barton, A. D.—(1953) *Exp. Cell Res.*, **5**, 147.
Moog, F.—(1946) *J. cell. comp. Physiol.*, **28**, 197.
*Idem* and Steinbach, H. B.—(1946) *Ibid.*, **28**, 209.
Novikoff, A. B., Podber, E. and Ryan, J.—(1950) *Fed. Proc.*, **9**, 210.
*Iidem* and Noe, E.—(1953) *J. Histochem. Cytochem.*, **1**, 27.
Pickels, E. G.—(1943) *J. gen. Physiol.*, **26**, 341.
Roche, J.—(1951) ‘The Enzymes’ 1, Part 1, 473. New York (Academic Press).
Schneider, W. C. and Hogeboom, G. H.—(1951) *Cancer Res.*, **11**, 1.
Slautterback, D. B.—(1953) *Exp. Cell Res.*, **5**, 173.
Tsuboi, K. K.—(1952) *Biochim. biophys. Acta*, **8**, 173.